# A qualitative study on the participation experience in a mental health recovery program based on WHO QualityRights in South Korea

**DOI:** 10.3389/fpsyt.2026.1782854

**Published:** 2026-04-16

**Authors:** Hanna Lim, Anna You, Jin Kim, Seongsu Kim, Yong Hyuk Cho, Shin Kwon Kim

**Affiliations:** 1 Department of Medical Humanities and Social Medicine, Ajou University School of Medicine, Suwon, Republic of Korea; 2 Department of Psychiatry, Ajou University School of Medicine, Suwon, Republic of Korea; 3 Department of Psychiatry, Ajou University Hospital, Suwon, Republic of Korea; 4 College of Nursing, Ajou University, Suwon, Republic of Korea; 5 Dawon Mental Health Clinic, Suwon, Republic of Korea; 6 Korean Open Dialogue Institute, Suwon, Republic of Korea; 7 Suwon Mental Health Welfare Center for Adults, Suwon, Republic of Korea

**Keywords:** WHO QualityRights, Open Dialogue, mental health recovery program, rights-based, person-centered, recovery-oriented, qualitative study

## Abstract

**Introduction:**

The World Health Organization’s QualityRights initiative offers a practical framework for developing rights-based, person-centered, and recovery-oriented mental health systems. In Korea, the face-to-face WHO QualityRights specialized training module, *Recovery practices for mental health and well-being*, was culturally and clinically adapted for local use, incorporating Open Dialogue principles. This adaptation led to the development of the group-based “QualityRights Recovery Program.” This study examines the experiences and perspectives of individuals with lived experience of mental health challenges, their family caregivers, and mental health practitioners who participated in this program to inform the local implementation of recovery-oriented mental health practices.

**Methods:**

Eighteen participants were recruited from two mental health facilities in Suwon, Republic of Korea. Researchers conducted semi-structured interviews and used thematic analysis to examine participants’ experiences with the 13-week QualityRights Recovery Program, which was adapted for the Korean clinical context.

**Results:**

Four major themes emerged: (1) participation and engagement in recovery, (2) changes in communication and decision-making, (3) mutual understanding and shifts in perception, and (4) redefining recovery concepts and therapeutic aims.

**Conclusion:**

Participants’ perspectives on the QualityRights Recovery Program indicate its potential to restore the autonomy and well-being of individuals with lived experience, while also positively influencing the perspectives of their caregivers and practitioners. These findings provide guidance for expanding rights-based, recovery-oriented mental health interventions in Korea.

## 1 Introduction

The United Nations Convention on the Rights of Persons with Disabilities (CRPD), adopted in 2006, marked a significant global development. The Republic of Korea ratified the treaty in 2008 (1). The CRPD aims to ensure that all persons with disabilities fully and equally enjoy all human rights and fundamental freedoms, allowing them to live with dignity and legal equality (2). In response, Korea enacted the Act on the Improvement of Mental Health and the Support for Welfare Services for Persons with Mental Illness (Mental Health Welfare Act) in 2017 (3), reflecting a national commitment to mental health reforms consistent with the CRPD.

Building on international and national developments, the WHO QualityRights Initiative offers a practical framework and strategy to support CRPD-ratified countries, such as Korea, in promoting mental health systems and services that respect human rights. QualityRights focuses on three core areas: capacity building, development of community-based services, and policy and legislative reform (4). To implement this framework, WHO developed two main tools. First, the QualityRights Toolkit, released in 2012, serves as an assessment tool to evaluate the compliance of mental health services with CRPD standards (5). In Korea, Park and Kahng (6) validated the Korean version of this toolkit among 348 psychiatric inpatients, identifying ways to create service environments that support human rights. Second, in 2019, WHO introduced QualityRights training materials for a range of stakeholders (e.g., advocacy groups, families, academics, health professionals, social workers, policymakers) to strengthen human rights-based, person-centered, and recovery-oriented mental health care (4, 7).

Following their launch, studies of these materials have shown both effects and implementation challenges. Duffy and Kelly (8) identified core modules relevant to Ireland’s mental health system and recommended legislative and workforce reforms. Mion et al. (9) reported that Brazilian healthcare professionals gained a deeper understanding of human rights and reduced stigma after training. Morrissey (10) observed 10–40% positive attitude changes after training among 27 participants (service providers, individuals with lived experience, and families), with the greatest improvements in views on involuntary treatment, independent living, legal capacity, and resource allocation. However, some experts debate the initiative’s effect, noting that it may be seen as unduly critical of mainstream psychiatry and overly restrictive regarding involuntary treatment (11), while others note its potential to support collaborative partnerships and improve the quality of mental health services (12).

Given these demonstrated benefits and ongoing international debates on QualityRights implementation, there is a growing movement in Korea to adopt WHO QualityRights-based programs. Leading institutions, such as Ajou University Hospital and Yongin Mental Hospital, have played a central role in initiating and advancing these efforts within the country (13, 14). For example, our research team at Ajou University Hospital piloted a program for people with lived experience, family caregivers, and mental health practitioners using a specialized WHO QualityRights training resource (15). This pilot revealed interpretive tensions similar to those found internationally. Specifically, we found that presenting recovery-oriented care in direct contrast to traditional clinical practice, or strongly emphasizing strengths-based over deficit-based approaches, could be seen as implicitly criticizing established psychiatric methods. These findings indicate the need to develop approaches that align the ideal values of QualityRights with the practical realities of everyday clinical work.

Following these initial applications, a national research project began in 2022 to systematically adapt the WHO QualityRights *Recovery practices for mental health and well-being* training resource to Korean clinical settings (16). This process resulted in the QualityRights Recovery Program, which preserved the core themes, values, and direction of the original material while making key changes: case examples were localized, and value-driven dichotomies were replaced with open-ended, empathic, and mentalization-focused discussions to support reflection on one’s own and others’ thoughts and feelings (17). Open Dialogue, an innovative approach that treats psychotic symptoms as meaningful communication rather than pathology through close collaboration with families and social networks (18), was also integrated throughout the program. This integration aimed to promote active participation and shared understanding, building on recent evidence of its feasibility in Korea (13). This study examines how participation in the QualityRights Recovery Program affects the experiences and perspectives of individuals with lived experience, their family caregivers, and mental health practitioners through in-depth interviews, offering insights into its practical implementation and feasibility in Korea.

## 2 Methods

### 2.1 Participants

Participants were purposively recruited from two mental health facilities in Suwon, Republic of Korea: a psychiatric day hospital (March–July 2024) and a community-based mental health center (June–September 2024). The full program cohort consisted of 56 participants enrolled in the QualityRights Recovery Program (26 from the day hospital and 30 from the mental health center). From this cohort, 18 participants agreed to participate in the qualitative phase. The interviewee group comprised eight individuals with lived experience, five family caregivers, and five mental health practitioners (see **Table 1** for demographic characteristics). All three stakeholder groups were included to examine psychological changes and the development of shared understanding across perspectives. Direct written informed consent was obtained from each interview participant prior to data collection. The study protocol was approved by the Institutional Review Board of Ajou University Hospital (AJOUIRB-SB-2024-397).

**Table 1:** Demographic characteristics of participants.

Participant	Gender	Age	Primary diagnosis	Duration of illness*	History of hospitalization
Individual with Lived Experience A	Male	21	Bipolar disorder	8 yrs	4 times
Individual with Lived Experience B	Female	28	Not disclosed	4 yrs	2 times
Individual with Lived Experience C	Male	33	Bipolar disorder	3 yrs	2 times
Individual with Lived Experience D	Female	55	Schizophrenia	20+ yrs	Unspecified
Individual with Lived Experience E	Male	55	Schizophrenia	20+ yrs	4 times
Individual with Lived Experience F	Female	29	Schizophrenia	3 yrs	Once
Individual with Lived Experience G	Female	54	Not disclosed	36 yrs	3–4 times
Individual with Lived Experience H	Female	27	Schizophrenia	1 yr	None
Participant	Gender	Age	Relationship to the individual	Diagnosis of the individual under care	Duration of care*
Family Caregiver A	Female	56	Mother	Bipolar disorder	10 yrs
Family Caregiver B	Male	58	Older brother	Schizophrenia	7 yrs
Family Caregiver C	Female	58	Mother	ADHD, Schizophrenia	15 yrs
Family Caregiver D	Male	63	Father	Schizophrenia	1 yr
Family Caregiver E	Female	57	Mother	Schizophrenia	7 yrs
Participant	Gender	Age	Job title	Years of experience	
Mental Health Practitioner A	Female	26	Social Worker	3 yrs	
Mental Health Practitioner B	Male	29	Social Worker	6 yrs	
Mental Health Practitioner C	Female	30	Social Worker	7 yrs	
Mental Health Practitioner D	Female	47	Mental Health Nurse	23 yrs	
Mental Health Practitioner E	Female	43	Mental Health Nurse	4 yrs	

*Based on the date of diagnosis.

### 2.2 Materials

This study used the QualityRights Recovery Program, adapted from the WHO QualityRights framework—specifically *Recovery practices for mental health and well-being*—to fit the Korean clinical context (15). During the initial implementation of the original WHO QualityRights materials, we observed that certain scenarios and examples developed for diverse global settings were not easily relatable to participants in the Korean context. In some instances, this was found to hinder engagement and concentration during group discussions. To improve contextual relevance and participant engagement, the materials were culturally adapted while maintaining the core principles and structure of the original program. Such contextual adaptation may also facilitate reproducibility in other settings with similar socioeconomic and mental health service contexts.

Specifically, original vignettes were replaced with culturally grounded Korean scenarios incorporating locally familiar names, domestic life experiences (e.g., illness onset during mandatory military service), and nuanced family communication patterns typical in Korean culture, such as indirect expressions of care and deference between parents and children. Additionally, selected multimedia materials were adapted to better support participants’ understanding of the program themes. Some of the original WHO video examples were replaced with culturally familiar Korean media content to provide a more relatable context for Korean participants. These modifications were agreed upon by a multidisciplinary group of experts—comprising psychiatrists, mental health nurses, social workers, and clinical psychologists—involved in the cultural adaptation process.

The 13-week program consisted of an initial orientation and 12 core thematic sessions, each lasting 90 to 180 minutes. A main facilitator led the sessions, supported by one or two co-facilitators who monitored participation and assisted attendees. Sessions included psychoeducation, group discussions, video-based materials, and experiential exercises to support mentalization and develop a shared perspective. Participants received printed handouts for individual or small group work, encouraging active engagement and reflection. To ensure diverse perspectives, participants were organized into small heterogeneous groups, with each group including a mix of individuals with lived experience, family caregivers, and mental health practitioners. Individuals with lived experience and family caregivers were assigned to separate groups to encourage open discussion, while mental health practitioners could join any group regardless of prior therapeutic relationships. All participants received a certificate of completion at the end of the program (see **Table 2** for details of the core topics).

**Table 2:** Session structure of the WHO QualityRights module and the QualityRights recovery program.

Original WHO module: *Recovery practices for mental health and well-being*	Adapted session title: QualityRights recovery program	Session content
1. What is recovery? 2. Recovery-oriented services and practices 3. Recovery focus on assets and strengths 4. Promoting hope 5. Values in recovery 6. Working alongside people 7. Boundaries within the context of recovery practices 8. Positive risks in recovery 9. Supporting people to reconnect with their communities 10. Communication skills 11. Recovery plans 12. Recovery Wheel	0. Orientation	Introducing the program and the concept of recovery
	1. What is recovery?	Understanding personal recovery and setting expectations
	2. Recovery-oriented approach	Introduction to recovery values and rights-based care
	3. Recovery-oriented mental health services and promoting hope	Promoting hope and exploring the role of community support
	4. Being present in the journey of recovery	Building empathy and shared responsibility
	5. Boundaries in the journey of recovery	Exploring personal limits and healthy relationship boundaries
	6. Positive risk-taking for recovery	Encouraging autonomy and growth through measured challenges
	7. Reconnection to community	Enhancing social participation and community reintegration
	8. Recovery communication skills 1	Foundational dialogue and active listening skills
	9. Recovery communication skills 2	Addressing stigma and difficult conversations
	10. The star of recovery	Empowering collaborative choices in care
	11. Recovery planning 1	Designing a personal recovery roadmap
	12. Recovery planning 2	Crisis planning and sustaining progress post-discharge

### 2.3 Data collection

Data were collected through semi-structured interviews conducted in Korean. These interviews were held primarily at program completion or 1–2 weeks before completion, in a private room at either the community mental health center or university hospital, depending on participants’ program sites. Each participant was interviewed once for approximately 60 minutes. The interview guide addressed experiences with the program, including motivations for participation, perceived effects, comparisons with other programs, changes in self-perception, communication, decision-making, interpersonal relationships, and understanding of recovery and treatment goals (see **Supplementary Tables 1a-c** for the detailed interview questions across all participant groups). Two researchers with prior qualitative research experience conducted all interviews. With participant consent, interviews were audio-recorded. Preliminary transcripts were produced using the online speech-to-text platform (*Clovanote*) and then meticulously reviewed by a research team member for verbatim accuracy. Data were anonymized using participant codes and securely stored on password-protected devices accessible only to the research team to ensure confidentiality.

### 2.4 Data analysis

We analyzed the data using the thematic analysis framework developed by Braun and Clarke (19). All verbatim transcripts were managed using Microsoft Excel, which facilitated a deeply engaged, line-by-line examination. Our analytical procedure unfolded in the following stages: First, through data familiarization, we read the transcripts repeatedly to grasp the nuances and context of the participants’ experiences. Second, initial coding was conducted by identifying and summarizing meaningful phrases. Third, we developed subthemes and themes independently for each participant group. To synthesize these perspectives, we mapped the findings into a thematic matrix, which allowed us to identify both unique insights and commonalities among the three participant groups. Ultimately, this process enabled us to move beyond group-specific findings and construct overarching themes that reflect the multidimensional nature of the program.

To ensure the analytical rigor of the study, we employed several verification procedures. Peer-debriefing was conducted through regular analytic discussions between the primary analyst (HL) and the auditor (AY) to review coding decisions, resolve interpretive discrepancies, and refine subthemes. For ambiguous interpretations, we employed triangulation by involving the primary program facilitator and developer of the Korean adaptation (YC) in the analytic discussions; this ensured that our interpretations remained grounded in the practical and cultural context of the intervention. Finally, a senior researcher (SKK) conducted a final analytic audit to confirm the logical derivation of themes from the raw data and to ensure that the findings accurately represented the participants’ perspectives.

All interviews were conducted, transcribed, and analyzed in Korean to preserve the original nuances of the participants’ voices. For the English manuscript, an initial draft was prepared using AI-assisted translation (*Perplexity*), followed by rigorous review by research team members with international academic backgrounds to ensure conceptual consistency. The final manuscript underwent professional language editing (*Editage*) to ensure linguistic accuracy and the precise articulation of our findings.

## 3 Results

Thematic analysis identified four major themes, each containing one subtheme for each stakeholder group (three subthemes per theme, totaling 12): Participation and engagement in recovery, Changes in communication and decision-making, Mutual understanding and shifts in perception, and Redefining recovery concepts and therapeutic aims (see **Supplementary Table 2** for detailed themes, subthemes, and supporting quotations).

Each participant is identified by an abbreviation for their group and an alphabetical label. For example, individuals with lived experience are labeled Individual A to H, family caregivers as Caregiver A to E, and mental health practitioners as Practitioner A to E.

### 3.1 Participation and engagement in recovery

This theme reflects the broad involvement of diverse stakeholders in the QualityRights Recovery Program, which provides a rare opportunity for tripartite dialogue. Individuals with lived experience expand self-agency through self-expression opportunities, family caregivers build empathetic connections while facing shared challenges, and mental health practitioners deepen professional understanding through ongoing multisession interactions.

#### 3.1.1 Individuals with lived experience: expanding self-expression and shared understanding through open discussion and presentation

Individuals with lived experience actively participated in open discussions and presentations, identifying themselves as key agents in their own recovery. This involvement strengthened their sense of agency and deepened self-understanding within a supportive, empowering environment. Participants described the experience as intellectually engaging and emotionally fulfilling, expressing satisfaction with their active role in the recovery process.

It was really beneficial because we could have discussions based on a lot of psychiatric research, which allowed us to talk in depth about our psychological aspects. It also made us reflect on certain concerns with each other. Overall, I think the biggest strength was that it felt comfortable. (Individual C).

It stimulates my thinking … it keeps me reflecting, and I think that’s really good. I really like that I get to present my thoughts, being encouraged to think and then share them, that’s what I liked. (Individual D).

#### 3.1.2 Family caregivers: connecting with others facing similar challenges and gaining new insights

Family caregivers gained emotional support and empathy by connecting with others facing similar challenges. While sharing their experiences sometimes caused emotional strain, developing mutual understanding with other caregivers provided psychological reassurance and comfort.

It was a time when I could listen to others’ thoughts as well as share my own. Of course, while talking about myself, I might have exposed some personal weaknesses, but these were stories I couldn’t talk about anywhere else. So naturally, when I talked with others who share the same empathy, I felt more comfortable and at ease. Also, by hearing about other people’s situations, facing even tougher challenges than mine, how they handle things, and what they think, I could compare their experiences with my own. So, for me, these moments became really meaningful. (Caregiver B).

Caregiver D reported communication barriers with his daughter, who has lived experience, and stated that the recovery program provided meaningful opportunities for connection and hope.

In my case, the way the program was conducted was really good. I had actually wanted something like this even before going to the day hospital. Because when I talk with my daughter at home, there are always limits—certain barriers that come up between us. But being able to talk with others, to see my daughter communicating with other people, and to present in that kind of space felt really good and promising to me. (Caregiver D).

#### 3.1.3 Mental health practitioners: professional growth through sustained multi-session engagement

Mental health practitioners expanded their professional insight and understanding by consistently listening to individuals with lived experience and their families across 12 sessions. Unlike typical single-session programs, this multi-session communal approach enabled deeper observation of participants’ evolving perspectives over time while creating a shared space transcending traditional counseling.

First of all, there was never a place where individuals with lived experience, practitioners, and family members could all gather together like this. I think the opportunity itself was meaningful … It was also a chance to think more deeply about what each person was thinking. In reality, you can’t get all that from counseling alone. But through this program, I feel I gained a much better understanding of what individuals and their families think. (Practitioner B).

While typical educational programs provide clear benefits even in a single short session, this 12-session program offered opportunities to consistently observe the same individuals with lived experience and caregivers over time and truly understand their perspectives. Reflecting on this, it really helped me appreciate the program’s substantial effectiveness. (Practitioner E).

### 3.2 Changes in communication and decision-making

This theme shows significant changes in communication and decision-making based on Open Dialogue principles. In the program’s dialogic, nonjudgmental environment, participants began to listen genuinely to one another, which encouraged self-reflection, respect for different perspectives, and empathic engagement with recovery challenges.

#### 3.2.1 Individuals with lived experience: positive change through communication and self-reflection

Participants with lived experience emphasized self-reflection, which broadened their perspectives and increased emotional openness. Several individuals referred to “positive risk,” which encouraged them to accept certain fears as necessary for progress. Engaging in casual conversation also reduced anxiety and supported freer self-expression.

What still comes to mind is the concept of “positive risk” I was quite impressed by it, I guess that to move forward, you have to accept some fear. That part was quite meaningful to me. (Individual B).

I’m generally not very good at having conversations. It’s not that I don’t want to talk to someone, but I worry about what kind of conversation we should have … but I realized that just talking for the sake of talking can be good too. Just talking can break the tension among people, so it’s okay to say silly things sometimes. I thought, “Maybe I should just say whatever comes to mind,” and I went to a peer supporter. Then, we kind of went back and forth like that (Individual F).

#### 3.2.2 Family caregivers: a process of decision-making that incorporates the individual’s voice

Family caregivers described a shift from a protective instinct to accepting risks and respecting individuals’ autonomy in decision-making. By supporting personal choices, they increased agency in those they cared for and enabled collaborative decision-making.

Last time, my son mentioned there’s some Language Day event, and said he was going to Seoul for it. I immediately felt scared when he said he was going to something like that … But then I remembered what we did in that program. I initially participated in the QR program hoping he would communicate more, but when he actually said he was going to the Language Day event, I started thinking, “Maybe it’s better just to stay safe than to expose him to risk.” But then I realized, “Sometimes I need to let him do it.” (Caregiver A).

Before, I used to focus everything on my own thoughts and act accordingly. But through this program, I learned that I shouldn’t do that. Instead, I need to focus on the individual and also adjust my words and actions accordingly. So now, when making decisions, instead of forcing or telling her what to do, I listen to her thoughts and let her make the decisions. Then, we work together based on those decisions. (Caregiver B).

#### 3.2.3 Mental health practitioners: from confrontation to empathetic listening in recovery support

Mental health practitioners gained greater experiential understanding of the challenges individuals with lived experience encounter during recovery, which led to changes in their perspectives and attitudes. They also moved away from traditional responses to hallucination experiences, such as confrontation and correction, and instead adopted approaches focused on listening and empathy.

The program was held in the morning, so some individuals with lived experience often arrived late despite our reminders the day before and close attention. This initially frustrated me, but through the program, I gained deeper insight into their genuine efforts to attend despite challenges they face—such as medication side effects making it hard to wake up—in their recovery journey. (Practitioner A).

Honestly, as a professional, it’s very difficult to empathize with experiences like auditory hallucinations in counseling. We tend to confront or try to correct those experiences. When we hear about hallucinations, we usually think the symptoms have worsened or sometimes suspect the person hasn’t taken their medication. But attending this program made me think that rather than doubting or pushing the person to take medication, simply listening, empathizing, and helping them organize their thoughts might be much more helpful for them. (Practitioner B).

### 3.3 Mutual understanding and shifts in perception

This theme shows a nuanced development in relationships: individuals became more aware of caregivers’ challenges and practitioners’ empathy, caregivers built trusting partnerships with practitioners, and practitioners recognized the need for tailored approaches to support families.

#### 3.3.1 Individuals with lived experience: a new beginning in relationships through understanding caregivers and practitioners

Individuals with lived experience reported that engaging with caregivers and practitioners led to new insights about relationships. Recognizing caregivers’ burdens and practitioners’ humanity broadened their perspectives.

There was a caregiver of a patient with more severe symptoms in my group, and I thought about how difficult it must have been to take on that role. It made me feel that I should receive treatment—or even just in daily life—build a good relationship with my parent. (Individual C).

I used to think nurses at the hospital were just people who gave directions or handled appointments, but seeing how they asked questions in such a friendly way felt different. I realized they could also be warm and approachable figures. That was surprising to me. (Individual H).

Not all individuals with lived experience interpreted these relationships similarly. Individual E emphasized the limits of professional knowledge and contrasted it with the deeper understanding that comes from lived experience.

Because the professionals have studied, they understand when we have our symptoms. But I once wrote something about this. Even if the professionals have studied and treat patients, they can’t fully understand the mind of an individual with lived experience. They listen and then prescribe or provide treatment. But for me, when a patient says what he or she is thinking, I can easily relate, thinking, “I’ve felt the same way.” (Individual E).

#### 3.3.2 Family caregivers: improved trust and positive perceptions toward practitioners

Family caregivers reported a gradual shift in their perceptions of mental health practitioners. While they initially saw practitioners as performing only practical duties, over time, they developed respect and appreciation for them.

At first, I thought of the practitioners simply in a practical way—like, they do this work as their job, and I meet them because of my family member with mental illness … But over time, I came to understand how important the practitioners’ role really is. They are true professionals with expertise. And I also came to see them as perhaps the only allies who can stand alongside families like ours and fight against social prejudice. My perspective shifted a great deal. (Caregiver C).

In addition, caregivers observed that practitioners intentionally built empathy and common ground with individuals with lived experience by interacting informally and inclusively in group settings. Caregivers perceived this approach as effective in increasing participation and engagement.

I’m really grateful to the social workers and nurses—these people really think from the individual’s perspective. They try to join in with us, creating a sense of empathy and common ground. Because of that, the individuals also respond and follow along … I don’t think that atmosphere would be possible if individuals with lived experience were only gathered by themselves. But since these practitioners sit together at the tables, one or two at a time, and interact like that, I think it’s very effective. (Caregiver D).

Caregivers observed practitioners’ ongoing concern and efforts to better support individuals with lived experience, which increased caregivers’ trust and appreciation of practitioners.

I realized that they, too, genuinely keep thinking about the individuals with lived experience, constantly, and that they have the heart to try to do even a little more for them. I could sense that the practitioners are always considering how they can be more helpful and putting effort into it. This time, I really felt that deeply. (Caregiver E).

#### 3.3.3 Mental health practitioners: recognizing the need for caregiver-centered, differentiated approaches

Practitioners reflected on their experiences working with caregivers during the program. They described challenges in meeting caregivers’ expectations and emphasized the importance of acknowledging caregivers’ own difficulties.

The caregivers who participated in the program were usually those who cared for their children very devotedly. But they would often demand so much from us. At first, I found that very difficult. I didn’t really understand their lives, but through the program, I was able to empathize more with their hearts and perspectives. And I also noticed that the caregivers themselves began to change through the program. Their attitude shifted toward one of more understanding of the individuals. Because of that change, they contacted me a lot less than before. It made me feel that the caregivers, too, were truly going through their own recovery process. (Practitioner A).

Practitioners also noted the need to address family challenges using differentiated, caregiver-centered support and to empathize with the emotions of both families and individuals.

In the past, I mostly approached families in a treatment-focused way, for example, asking them to help their sons or daughters take their medication consistently. But through the program, I came to realize that the families themselves are struggling, and I learned what kind of thoughts and feelings they have while dealing with their loved ones … Now I understand that their perspective is different from the individual’s perspective, and of course, the approach should differ accordingly … When speaking with families, we shouldn’t only talk about the individual; we also need to listen to and empathize with the family’s own emotions. (Practitioner B).

### 3.4 Redefining recovery concepts and therapeutic aims

This theme shows a shift in how recovery is understood by individuals with lived experience, family caregivers, and mental health practitioners. Recovery is not seen as a cure or complete independence, but as an ongoing, relational, and value-driven process that occurs in daily life. Each group describes its own way of redefining goals, hopes, and roles within this process.

#### 3.4.1 Individuals with lived experience: embracing one’s true self and seeking meaning in life as a new paradigm of recovery

Recovery is now recognized as a personal process focused on living authentically and finding meaning, even when challenges persist, rather than simply the absence of symptoms. This perspective emphasizes building a stable, fulfilling life that aligns with one’s sense of self, extending beyond clinical improvement.

After participating this program, I think I developed a treatment goal. Taking medication is to prevent worsening, but for real improvement, it’s about meeting someone, doing good things with that person, and finding something I like to do for myself. I think that has become my recovery goal now. (Individual C).

To me, recovery is simply a sense of inner stability. Just being able to live … The program facilitator explained the idea of a sense of self. I thought, “Yes, that’s me.” I had been living with a lot of masks on, and that was not easy … The idea of being able to just live as myself was very helpful. Even if I hear voices or have some illness, just living in my own way … (Individual F).

#### 3.4.2 Family caregivers: redefining recovery and shifting hopes for the progress of individuals with lived experience

Families may initially view recovery as an endpoint, such as a complete cure or full independence. However, as they observe the ongoing process, they begin to recognize incremental progress and daily stability as important milestones in recovery.

I think medication can have some effect to a certain extent now, but for those aspects that medication cannot address, I believe the most important thing is the mutual emotional connection … When my sister is quiet or reserved, I initiate conversation, encourage her to share everyday stories, and ask questions … (Caregiver B).

I used to think that recovery meant my son stopping medication, living like a typical person with a job and family—basically 100% discharged from a psychiatric hospital. But I’ve come to realize that recovery isn’t reaching a mountain peak. Recovery is a process, a process that needs support … So, in a way, I feel grateful that my son is now able to stay away from many risks, control his eating, and has even expressed a desire to hike mountains. Sometimes he sends me pictures from the mountains, showing me that he’s recovering. If his recovery means this, even if he doesn’t reach the goals of having a home and job that I once hoped for, I would be truly content. (Caregiver E).

#### 3.4.3 Mental health practitioners: transforming support and roles and everyday interactions

Practitioners are experiencing a paradigm shift in their roles. Rather than only providing guidance or focusing on clinical targets, they now prioritize attentive listening, support autonomy, and engage with individuals’ hopes and goals. This change has been driven by practitioners’ reflections from participating in the program, indicating a shift toward person-centered, collaborative practice.

I’ve been reflecting on my approach to recovery. While I’ve spent a lot of time discussing the current symptoms and conditions of individuals with lived experience, I realized that I may not have truly explored their dreams for the future. I tended to focus on saying, “I hope you can move forward like this,” but I didn’t really ask, “What do you actually want to achieve, and how far do you want to go?” (Practitioner D).

I used to put a lot of emphasis on the educational aspect of my work, but now I feel that I’ve become more open to simply listening and understanding what this person is thinking and what they actually want to do. I think I’ve gained some flexibility in my thinking. There was always a sense of duty and responsibility, a feeling that I had to solve their problems for them. But I’ve started to realize that I don’t always have to choose their path for them. (Practitioner E).

## 4 Discussion

This study examined the experiences of individuals with lived experience of mental health challenges, family caregivers, and mental health practitioners who participated in the group-based QualityRights Recovery Program. Adapted from a WHO QualityRights training module to fit local contexts, the program also incorporated Open Dialogue principles to encourage more dialogical and collaborative practices. Delivered over 13 weeks in clinical settings, its impact was assessed through in-depth interviews to determine how it supports recovery-oriented practice and its relevance in contemporary Korean mental health services.

Recovery-oriented practice is most effective when grounded in human rights and agency (20). However, in clinical settings, the characteristics of mental illness—especially psychotic and negative symptoms—often lead to prioritizing the perspectives of caregivers and practitioners over those of individuals with lived experience (21, 22). This tendency is prevalent across various cultural contexts; in South Korea, for example, the views of individuals with lived experience are frequently overshadowed by those of family members and practitioners in decision-making (23–26). Similarly, in Ghana, a strong endorsement of paternalistic attitudes often leads to family members providing informed consent without seeking direct input from individuals themselves (27). These shared observations underscore the importance of addressing the power imbalance in clinical settings to achieve care that is truly centered on individuals with lived experience. In this context, the present discussion organizes findings into four main themes, each examined from the perspectives of the three participating groups. These themes show that reclaiming and amplifying the voices of individuals with lived experience is essential for advancing recovery-oriented clinical practice.

The first theme, “Participation and engagement in recovery,” shows that collaboration among individuals with lived experience, family caregivers, and mental health practitioners provides therapeutic benefits by empowering all stakeholders. Participants reported that opportunities for these groups to meet and discuss were scarce. However, when such gatherings occurred, individuals with lived experience appreciated the chance to express themselves and share their perspectives, caregivers connected with others facing similar situations, and practitioners gained insights through group discussions that extended beyond individual counseling. Tjaden et al. (28) found that resource groups including patients, families, friends, and professionals who set recovery goals together enhance personal recovery and empowerment. Although Chung and Yang (29) described efforts to introduce WHO QualityRights into Korean mental health services, their focus was limited as caregivers were not included in the program. By contrast, this study found that bringing together all three groups was valuable, as their direct interactions led to greater mutual understanding and cooperation. Strengthening these collaborative structures in Korean services, where all groups can participate actively, is essential for empowering participants and supporting ongoing recovery-oriented care.

The second theme, “Changes in communication and decision making,” examines improvements in communication styles and decision-making processes as mutual understanding increases among program participants. These positive changes occurred because the program intentionally incorporated Open Dialogue principles into the WHO QualityRights framework. These principles included social network perspective, responsibility, tolerance of uncertainty, and dialogism (30, 31). Von Peter et al. (32) state that Open Dialogue encourages active, creative exchanges of diverse viewpoints rather than only seeking consensus. The facilitator played a key role by balancing strong emotional expressions from caregivers and practitioners’ focus on corrective strategies, while encouraging individuals with lived experience to participate in dialogue. This dialogic approach was further operationalized through a closing circle at the end of each session, where every participant shared at least one sentence about the session. These processes led to meaningful changes for all participant groups. Individuals with lived experience made efforts to engage in dialogue, even when uncertain about what to say. Through this process, they shifted from seeing themselves as passive recipients of treatment to active agents in their own recovery. Caregivers began to value decisions that reflect the perspectives of individuals with lived experience. Practitioners moved from symptom-focused, corrective approaches to attitudes based on empathy and listening, appreciating individuals’ narratives.

The third theme, “Mutual understanding and shifts in perception,” highlights how reciprocal understanding led stakeholders to develop new views of each other. Psychoeducation on human rights, recovery, and person-centered care may represent an important starting point for reducing stigma and changing perceptions toward individuals with mental illness. Funk et al. (33) demonstrated that even self-paced WHO QualityRights e-training can lead to significant positive shifts in attitudes and reduce stigma among diverse stakeholders globally. However, educational approaches alone may not be sufficient to transform interpersonal understanding within everyday clinical contexts. In this regard, the present program provided structured opportunities for individuals with lived experience, family caregivers, and practitioners to engage in face-to-face intergroup dialogue around recovery-related themes and shared cases drawn from the WHO QualityRights framework, using culturally grounded fictional characters that allowed participants to discuss experiences in a safe and relatable manner. Through these interactions, participants were able to reflect on prior assumptions and develop deeper mutual understanding of each other’s perspectives. This approach demonstrates the therapeutic value of mentalization—the ability to understand behavior in oneself and others by considering thoughts, feelings, and intentions (34, 35). As participants engaged in these dialogues, they questioned previous assumptions and adopted new perspectives. Individuals with lived experience recognized caregivers’ burdens and practitioners’ empathy, while caregivers came to value practitioners as supportive partners. Practitioners acknowledged the need for more family-centered approaches. This reflects the caregiving burdens and decision-making challenges described in Huang et al. (36). Practitioners also embraced a more collaborative role, positioning themselves alongside individuals with lived experience and family caregivers to form a unified support network. Through this process, stakeholders understood that multiple perspectives can coexist within the same situation, revealing the limits of one’s assumptions.

**Figure 1:**
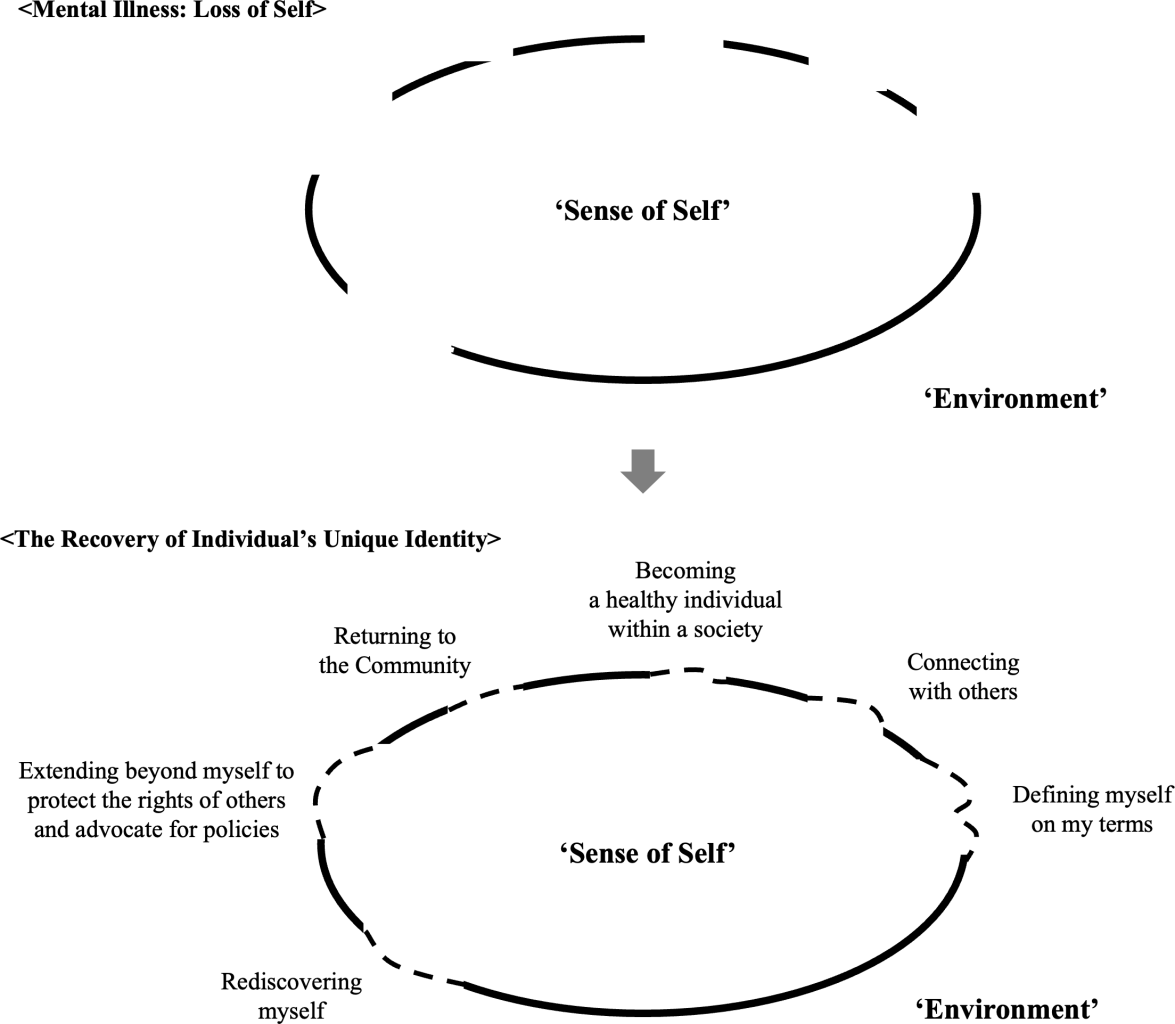
From loss to recovery of sense of self.

The fourth theme, “Redefining recovery concepts and therapeutic aims,” addresses the evolving view of recovery in mental health as a holistic process that extends beyond symptom reduction, as supported by Ponce-Correa et al. (37). This perspective includes embracing authentic self-identity, building meaningful connections, and transforming support to help individuals live fulfilling lives despite ongoing challenges. To make this approach practical, the program focused on the recovery journey, translating the abstract idea of recovery into concrete, actionable steps. From the first psychoeducation session, participants learned that mental illness often involves a loss of “sense of self” (20), and that recovery involves reclaiming these essential aspects of identity and autonomy (see **Figure 1**). The program presented interconnected topics across Sessions 1–12 to show a structured, integrated pathway toward these recovery domains. As the program progressed, all stakeholders recognized that the recovery journey creates a synergy greater than the sum of its parts. Recovery of self is ultimately understood as a shared journey, encompassing not only individuals with lived experience but also caregivers and practitioners.

These insights from the program exemplify the paradigm shift in mental health care. Traditionally, mental health care has been dominated by a disease paradigm. Its primary goal has been symptom remission—restoring normal bodily functions—as the main goal through cross-sectional assessments of physiological problems. This approach tends to overlook long-term health patterns like chronic conditions and risk buildup, ultimately reducing health to a mere absence of pathology (38). In contrast, the health paradigm views health as the ability to maintain balance through life’s changes. This includes adapting to challenges over time, regaining control, meaning, social roles, and resilience—even with ongoing symptoms (39). Recovery evolves as self-transformation—accepting suffering, fostering self-efficacy via autonomy and competence, and aligning purpose amid vulnerability (40, 41). The QualityRights Recovery Program bridges this shift in Korean clinical settings, culturally adapting rights-based, dialogic practices to empower individuals with lived experience, families, and practitioners toward holistic well-being.

A key strength of this study is its cultural and clinical adaptation of the WHO QualityRights *Recovery practices for mental health and well-being* training module (15) for Korean clinical settings. In line with Jeong and Lee (14), it promotes rights-based training and inpatient social support. Recruiting participants from both a university hospital and a community-based mental health center allowed examination of the program’s effects across different clinical settings. However, several limitations should be acknowledged. First, the relatively small sample size and the heterogeneity of participants—including individuals with lived experience, family caregivers, and mental health practitioners—may limit the transferability of the findings. While we organized our results to preserve the distinct voice of each stakeholder group within overarching themes, some group-specific nuances may be less pronounced than in studies with more homogeneous samples. Yet, the inclusion of multiple stakeholder perspectives allowed the study to capture recovery as a relational and dialogical process, which is central to both the QualityRights framework and Open Dialogue principles. Second, while the study spanned both hospital and community contexts, participants were self-selected and recruited from two urban clinical settings, which may reflect relatively high motivation toward recovery-oriented practices. This may limit the applicability of the findings to other geographical or less recovery-oriented contexts. Nevertheless, participants with such engagement were also particularly well positioned to provide in-depth reflections on their experiences with the program. Third, this study relied exclusively on qualitative data. The absence of longitudinal follow-up or complementary quantitative measures limits the ability to assess the long-term sustainability of the observed changes or to provide statistically generalizable evidence. Future studies involving larger samples, as well as longitudinal and mixed-method designs, would further strengthen the evidence base for recovery-oriented interventions. Despite these limitations, the rich qualitative data provide valuable insights into how recovery-oriented and rights-based practices are experienced by different stakeholders within a real-world clinical context.

## 5 Conclusion

This study highlights that while individuals with lived experience, families, and practitioners all strive for recovery, their differing perspectives can sometimes create unintended gaps in the recovery process. To bridge these gaps, meaningful recovery must be built on a shared foundation of open dialogue and collaborative goal-setting. Following the WHO QualityRights approach, this model adopts a right-based and recovery-oriented perspective, moving away from traditional control-centered practices toward a respectful partnership that honors individual choice and agency. Such engagement creates a space for individuals to rediscover who they are, find their own voice, and feel a true sense of belonging. Ultimately, these shared experiences help people rebuild a steady ‘sense of self,’ allowing them to define their own lives and lead their own recovery journeys. Based on these findings, we recommend that mental health policies prioritize co-participatory, rights-based programs to shift from provider-led interventions to person-centered support systems. Moving forward, clinical practice should shift from a top-down approach toward fostering a genuine partnership, where shared decision-making becomes the daily standard rather than an exception. This ensures that recovery is navigated together, valuing the individual’s voice as much as professional expertise.

## Data Availability

The original contributions presented in the study are included in the article/**Supplementary Material**. Further inquiries can be directed to the corresponding authors.
