# Supplementary material for: A qualitative study on the participation experience in a mental health recovery program based on WHO QualityRights in South Korea
Source: Front Psychiatry. 2026 Apr 16;17:1782854. doi: 10.3389/fpsyt.2026.1782854 (PMC13128598; doi:10.3389/fpsyt.2026.1782854)
Supplement: Supplementary file 1 [file DataSheet1.pdf]

Interview Questions by Participant Group

Supplementary Table 1a. Interview Questions for Individuals with Lived Experience

Category	**Lead Questions**	**Probe Questions**
Initial Experience	Could you describe the period when you were first diagnosed?	• What were the major life stressors at that time?<br>• What led you to your first hospital visit?<br>• What was your hospitalization experience like?
Post-discharge Life (If applicable)	How has your daily life been since being discharged from the hospital?	• How do you feel about living at home now?<br>• How is your relationship with your family and friends?<br>• Are there any specific concerns or positive moments in your daily routine?
Program Experience	What motivated you to participate in the QualityRights Recovery program, and what were your first impressions?	• What has it been like for you to participate in this program?<br>• How would you compare this program with other programs you have experienced, if any?
Changes & Impact	What changes have you noticed in yourself since participating in the program?	• Has your way of speaking or communicating changed?<br>• Have you noticed any changes in how you make decisions for yourself?<br>• Have you noticed any changes in your interactions or relationships with caregivers and practitioners?
Perception of Recovery	How has your understanding of 'recovery' changed through this program?	• How did you define recovery before the program compared to now?<br>• Has your personal treatment goal changed in any way?
Future Plans	Do you have any specific plans or hopes for the future?	• What motivated you to make these specific plans?<br>• What do you feel is the most necessary element for your life right now?

Supplementary Table 1b. Interview Questions for Family Caregivers

Category	**Lead Questions**	**Probe Questions**
Initial Experience	Could you share your experience as a caregiver when the individual was first diagnosed?	• What were the major stressors the individual faced at that time?<br>• How did you feel when the individual was hospitalized?<br>• How did the diagnosis affect the overall family atmosphere?
Daily Life (If applicable)	How have your life and the individual's been since the discharge?	• How do you feel about the individual living at home now?<br>• Are you able to maintain your own daily routines or work?

		• What are your primary concerns or joys in daily life?
Program Experience	What are your thoughts on your (or the individual's) participation in this program?	• What motivated the participation in this specific program?<br>• Which part of the program (e.g., discussions, presentations) was most memorable?
Changes & Impact	Have you noticed any changes in the interactions or relationships between you, the individual, and the practitioners?	• Do you find yourself allowing the individual more autonomy in decision-making?<br>• Have you noticed any changes in the individual's behavior or your relationship with them?<br>• In what ways has your understanding of the individual deepened?
Perception of Recovery	Has your perspective on the individual's 'recovery' shifted?	• What was your previous goal for the individual's treatment?<br>• How do you envision 'recovery' for the individual now?

Supplementary Table 1c. Interview Questions for Mental Health Practitioners

Category	**Lead Questions**	**Probe Questions**
Professional Context	Could you briefly describe your current role and professional experience?	• How long have you been working in this field?<br>• What are the most rewarding and challenging aspects of working with individuals and families?
Program Evaluation	What were your overall impressions of the QualityRights Recovery program?	• In what ways, if at all, does this program compare to your previous experiences with conventional rehabilitation?<br>• How did you perceive the dialogue- and presentation-heavy structure of the program?
Changes & Perception	Have you noticed any changes in the interactions or relationships with individuals and their caregivers?	• Did your understanding of the individuals or their caregivers change after the program?<br>• What was the most significant change you observed in the participants?<br>• Have there been any shifts in your professional philosophy regarding 'recovery'?
Professional Growth	How has this program influenced your attitude or behavior as a practitioner?	• Have you discussed this program with other colleagues?<br>• Are there specific approaches from this program you intend to apply in your future practice?
