# Supplementary material for: A qualitative study on the participation experience in a mental health recovery program based on WHO QualityRights in South Korea
Source: Front Psychiatry. 2026 Apr 16;17:1782854. doi: 10.3389/fpsyt.2026.1782854 (PMC13128598; doi:10.3389/fpsyt.2026.1782854)
Supplement: Supplementary file 2 [file DataSheet2.pdf]

**Supplementary Table 2.** Themes of Participation Experiences in QualityRights Recovery Program

| Themes | Subtheme | Category | Supporting Quotations (Example) |
|---|---|---|---|
| **Participation and Engagement in Recovery** | Individuals with Lived Experience:<br><br>Expanding self-expression and shared understanding through open discussion and presentation | Active Expression | - "I don't really hate presenting that much either, and I like writing, so it was good." (ILE B)<br>- "I love having to present my own thoughts." (ILE D)<br>- "They told us to write down our own thoughts. And then present when told... I think the program is pretty good." (ILE E) |
| | | Connecting through Interaction | - "Even if I couldn't speak, it felt like someone else would fill in for me, so it was reassuring." (ILE C)<br>- "By listening to various people's stories, I got to realize, 'Oh, that person thinks that way.'" (ILE D)<br>- "I think it was good to share my story and listen to others' stories." (ILE G) |
| | | Safe Dialogue Space | - "I think the biggest strength was that it felt comfortable." (ILE C)<br>- "The center director saw me positively. That's what I liked about it." (ILE E)<br>- "They waited for me to speak and listened well to what I said, so it was actually good." (ILE G) |
| | | Cognitive Stimulation | - "Taking positive risks... It was impressive that to move forward, you have to embrace even that fear." (ILE B)<br>- "Using my head repeatedly helped me focus without other thoughts getting in the way." (ILE C)<br>- "It stimulates my thinking … it keeps me reflecting, and I think that's really good." (ILE D) |
| | Family Caregivers:<br><br>Connecting with others facing similar challenges and gaining new insights | Emotional Ventilation | - "… these were stories I couldn't talk about anywhere else … when I talked with others who share the same empathy, I felt more comfortable and at ease." (FC B)<br>- "It felt comfortable because it was a place where people with the same pain gathered to share." (FC C) |
| | | Expanded Understanding | - "The process of learning about other people's situations and comparing them with my own thoughts was meaningful." (FC B)<br>- "It was an experience that allowed me to understand the positions of the individuals with lived experience, other caregivers, and practitioners." (FC E)<br>- "What sticks in my memory most is that the perspectives and thoughts of the individuals with lived experience were different from what I had expected." (FC F) |
| | | Practical Caregiving Insights | - "I received practical help in terms of how to communicate with the individuals with lived experience." (FC B)<br>- "I liked that they provided examples of how to handle emergency situations and gave us a chance to think them through." (FC D)<br>- "I was able to learn situational coping methods for when I deal with the individuals with lived experience." (FC F) |
| | Mental Health Practitioners:<br><br>Professional growth through sustained multi-session engagement | Experiential Awareness | - "I used to think I knew the individuals' feelings well, but I realized I didn't know everything and reflected on that." (MHP D)<br>- "I always approached things thinking there was an answer, but realizing there isn't felt like a wake-up call." (MHP E) |
| | | Sustained Interaction | - "Seeing people participate voluntarily until the end, I felt the power of this program." (MHP D)<br>- "This 12-session program offered opportunities to consistently observe the same individuals with lived experience and caregivers over time and truly understand their perspectives." (MHP E) |

| Themes | Subtheme | Category | Supporting Quotations (Example) |
|---|---|---|---|
| | | Meaningful Burden | - “Feeling moved, angry, and emotionally drained, but it’s meaningful burnout that leads to reflection.” (MHP B)<br>- “The pressure to get individuals with lived experience more involved and waiting for them to speak was challenging.” (MHP D) |
| **Changes in Communication and Decision-Making** | Individuals with Lived Experience:<br><br>Positive change through communication and self-reflection | Communication Competence | - “I’ve come to listen to other people’s opinions with more respect.” (ILE D)<br>- “I realized that just talking for the sake of talking can be good too. Just talking can break the tension among people, so it’s okay to say silly things sometimes.” (ILE F)<br>- “I became more active in conversations, and my way of talking changed in a positive way.” (ILE G) |
| | | Self-awareness & Agency | - “In the past I felt swept along by others, but now I see that not losing myself is important.” (ILE F)<br>- “Things about myself became organized, and I came to know what I like.” (ILE G)” |
| | | Positive Outlook | - “What still comes to mind is the concept of “positive risk” … I guess that to move forward, you have to accept some fear.” (ILE B)<br>- “I started to feel confident and resolved to keep a positive mindset.” (ILE G) |
| | Family Caregivers:<br><br>A process of decision-making that incorporates the individual’s voice | Person-Centered Partnership | - “I wait without invading his space until he comes forward on his own.” (FC A)<br>- “When making decisions, instead of forcing or telling her what to do, I listen to her thoughts and let her make the decisions. Then, we work together based on those decisions.” (FC B)<br>- “Previously I was cautious and watched his reactions, but now I treat him comfortably as adult to adult.” (FC C) |
| | | Empathetic Communication | - “When I listened without dismissing stories like auditory hallucinations, she started following me much more.” (FC B)<br>- “As we got to know each other better, we stopped arguing and our communication became more flexible.” (FC D)<br>- “Instead of forcing, I negotiate by asking ‘What good things will happen if you do this?’” (FC E) |
| | | Perspective Taking | - “I came to understand her auditory hallucinations not as ‘nonsense’ but as a ‘phenomenon’ to accept.” (FC B)<br>- “Instead of just telling them to stay home, I understood his fear of new challenges.” (FC E) |
| | Mental Health Practitioners:<br><br>From confrontation to empathetic listening in recovery support | Multi-dimensional Understanding | - “I could discuss experiences like hospitalization that individual counseling couldn’t cover, and it was helpful.” (MHP C)<br>- “I broke out of thinking only from the practitioner’s perspective and learned how individuals with lived experience and caregivers think.” (MHP E) |
| | | Listening over Intervention | - “Attending this program made me think that rather than doubting or pushing the person to take medication, simply listening, empathizing, and helping them organize their thoughts might be much more helpful for them” (MHP B)<br>- “I learned that during counseling, it’s okay not to always provide answers—sometimes just listening is beneficial enough.” (MHP E) |
| | | Beyond Assumptions | - “I hadn’t understood the difficulties in the individuals’ recovery journey before, but now I realize those challenges.” (MHP A)<br>- “I saw aspects of the client that aren’t visible in counseling, and realized that being silent doesn’t mean they have no thoughts.” (MHP B) |

| Themes | Subtheme | Category | Supporting Quotations (Example) |
|---|---|---|---|
| **Mutual Understanding and Shifts in Perception** | Individuals with Lived Experience:<br><br>A new beginning in relationships through understanding caregivers and practitioners | Relational Reframing with Caregivers | - "I tried initiating conversation with my dad first, setting aside my hurt feelings." (ILE B)<br>- "There was a caregiver of a patient with more severe symptoms in my group, and I thought about how difficult it must have been to take on that role." (ILE C)<br>- "I discovered a new side of my dad, who used to only give commands and instructions, as I saw him making efforts among people." (ILE H) |
| | | Humanized Recognition of Practitioners | - "It helped me understand practitioners in the same way I understand my own caregiver's feelings." (ILE C)<br>- "I came to humanly understand that even practitioners can't fully know the feelings of individuals with lived experience." (ILE D)<br>- "I used to think nurses at the hospital were just people who gave directions or handled appointments, but seeing how they asked questions in such a friendly way felt different." (ILE H) |
| | Family Caregivers:<br><br>Improved trust and positive perceptions toward practitioners | Re-valuing Partnership with Practitioners | - "I newly realized the stress and difficulties practitioners face dealing with mentally distressed individuals every day." (FC B)<br>- "At first, I thought of the practitioners simply in a practical way … But over time, I came to understand how important the practitioners' role really is." (FC C) |
| | | Collaborative Synergy | - "I don't think that atmosphere would be possible if individuals with lived experience were only gathered by themselves … since these practitioners sit together at the tables … I think it's very effective." (FC D)<br>- "I realized that … they(practitioners) have the heart to try to do even a little more for them(individuals)" (FC E) |
| | Mental Health Practitioners:<br><br>Recognizing the need for caregiver-centered, differentiated approaches | Emotional Resonance with Caregivers | - "I didn't really understand their(caregivers') lives, but through the program, I was able to empathize more with their hearts and perspectives." (MHP A)<br>- "I learned what kind of thoughts and feelings they(caregivers) have while dealing with their loved ones..." (MHP B) |
| | | Critical Self-Reflection about Caregivers | - "I realized that my repetitive focus on clinical directives—like hospital admissions or medication adherence—may have been presumptuous." (MHP B)<br>- "I realized I hadn't deeply considered caregivers' perspectives and felt the need to empathize with their feelings beyond just focusing on the individual." (MHP E) |
| **Redefining Recovery Concepts and Therapeutic Aims** | Individuals with Lived Experience:<br><br>Embracing one's true self and seeking meaning in life as a new paradigm of recovery | Self-Acceptance & Subjectivity | - "Recovery isn't about becoming like others but accepting myself and living as my true self." (ILE B)<br>- "I realized that doing what I want to do greatly helps my recovery." (ILE E)<br>- "The idea of being able to just live as myself was very helpful. Even if I hear voices or have some illness, just living in my own way..." (ILE F)<br>- "I feel like I'm leading my daily life on my own, not being controlled by medication." (ILE G) |
| | | Agency-Based Goal Setting | - "For real improvement, it's about meeting someone, doing good things with that person, and finding something I like to do for myself. I think that has become my recovery goal now." (ILE C)<br>- "I would like to make stability concrete and set productive goals like part-time jobs or working at a café." (ILE G)<br>- "I want to work a face-to-face part-time job to improve my social skills." (ILE H) |
| | Family Caregivers: | Reframing Recovery | - "I used to think recovery meant becoming 'normal' like others, but I realized recovery is a process in itself." (FC D) |

| Themes | Subtheme | Category | Supporting Quotations (Example) |
| --- | --- | --- | --- |
| | Redefining recovery and shifting hopes for the progress of individuals with lived experience | | - "I accepted that it's about living together with lifelong management, and this realization helped me move beyond depression." (FC E) |
| | | Differentiation | - "I realized that instead of overly focusing on the individual, it's better for the family to live healthy lives for everyone's sake." (FC A)<br>- "Separating myself from the individual, prioritizing my own retirement and stability is important for the whole family's well-being." (FC C) |
| | | Incremental Empowerment | - "Medication can have some effect to a certain extent … but I believe the most important thing is the mutual emotional connection ... I initiate conversation, encourage her to share everyday stories…" (FC B)<br>- "Setting specific treatment goals, monitoring progress, and aiming for small achievements…" (FC D) |
| | Mental Health Practitioners:<br><br>Transforming support and roles and everyday interactions | Symptom-Free to Functional Living | - "I realized that complete cure doesn't really exist—recovery is about taking medication well, maintaining daily life, and reducing symptoms." (MHP B)<br>- "It's not about symptoms completely disappearing but managing them like diabetes while maintaining social life—that's what recovery means now." (MHP C) |
| | | Authentic Engagement | - "I started having more everyday conversations rather than professional directives and approaching them like friends greatly improved rapport." (MHP B)<br>- "I developed the capacity to intervene less and wait for the other person to fully express themselves." (MHP E) |
| | | Expert-Led to Person-Centered | - "I realized I shouldn't impose my own definition of 'recovery' on individuals with lived experience." (MHP A)<br>- "I've become more open to simply listening and understanding … what they actually want to do … I've started to realize that I don't always have to choose their path for them." (MHP E) |

*Note. ILE=Individual with Lived Experience; FC=Family Caregiver; MHP=Mental Health Professional.
